# L-765,314 Suppresses Melanin Synthesis by Regulating Tyrosinase Activity

**DOI:** 10.3390/molecules24040773

**Published:** 2019-02-21

**Authors:** Jinhwan Kim, Yo-Han Kim, Seunghyun Bang, Hanju Yoo, InKi Kim, Sung Eun Chang, Youngsup Song

**Affiliations:** 1 Department of Biomedical Sciences, University of Ulsan, College of Medicine, Asan Medical Center, Olympic-ro 43-gil 88, Songpa-Gu, Seoul 05505, Korea; ailurophilee@gmail.com (J.K.); kimyohan0627@naver.com (Y.-H.K.); 2 Bio-Medical Institute of Technology (BMIT), Seoul 05505, Korea; bangsh1037@gmail.com (S.B.); julia_yoo@hanmail.net (H.Y.); 3 Department of Dermatology, University of Ulsan College of Medicine, Asan Medical Center, Olympic-ro 43-gil 88, Songpa-Gu, Seoul 05505, Korea; 4 Asan Institute for Life Sciences, Asan Medical Center, Seoul 05505, Korea; ik.kim@amc.seoul.kr

**Keywords:** L-765,314, protein kinase C, tyrosinase activity, depigmenting agents

## Abstract

Although melanin production is a key self-defense mechanism against ultraviolet radiation (UVR)-induced skin damage, uneven or excessive deposition of melanin causes hyperpigmentary disorders. Currently available whitening agents are unsatisfactory because of issues with efficacy and safety. To develop more effective depigmenting agents, we performed high-throughput melanin content assay screening using the B16F10 melanoma cell line and identified L-765,314 as a drug that suppressed melanin production in cultured melanocytes in a dose-dependent manner as well as cAMP- or 12-*O*-tetradecanoylphorbol 13-acetate (TPA)-stimulated melanin production without cytotoxicity. Interestingly, melanogenic gene expression was not altered by L-765,314. Rather, diminished melanin production by L-765,314 appeared to be caused by downregulation of tyrosinase activity via inhibition of protein kinase C (PKC). Because L-765,314 did not show any adverse effect in melanocytes, altogether our data suggest that L-765,314 could be a potential therapeutic candidate for skin hyperpigmentary disorders and further discovery of selective inhibitors targeting PKC might be a promising strategy for the development of depigmenting agents to treat hyperpigmentary disorders.

## 1. Introduction

Skin tissues play a major role in defense against a wide range of environmental threats. In their position at the forefront of our body’s defenses, skin tissues are directly exposed to ultraviolet radiation (UVR); repeated UVR exposure increases the risk of carcinogenesis via the induction of DNA damage and mutation [1]. Melanin is produced by skin tissues as a self-protection mechanism and serves as a natural absorbent of free radical species as well as a physical barrier to UVR [2].

Although the majority of melanin visible in the epidermis is accumulated in keratinocytes, its synthesis actually takes place in specialized melanin-producing cells, i.e., melanocytes. Melanocytes contain lysosome-related organelles, termed melanosomes, within which a series of enzymatic and non-enzymatic chemical reactions produces and determines the quantity and quality of melanin. The first and rate-limiting step of melanin synthesis is catalyzed by tyrosinase. Tyrosinase is produced by oxidation of l-tyrosine to l-dihydroxyphenylalanine (l-DOPA) and dopaquinone, which are precursors for melanin synthesis. Subsequently, l-DOPA is converted to either pheomelanin or eumelanin, depending on its reaction with cellular cysteine or dopachrome tautomerase (DCT) and tyrosinase-related protein 1 (Tyrp1). Finally, after completion of melanin synthesis, melanocytes transport melanin-loaded melanosomes to neighboring keratinocytes, which constitute around 90% of the basal layer of the epidermis and shield skin tissues from UVR damage.

While melanogenesis is a beneficial process to safeguard from external hazards, excessive or irregular deposition of melanin may cause unfavorable hyperpigmentary disorders, such as melasma lentigines and post-inflammatory hyperpigmentation, which are often recurrent and refractory to various treatments. Thus, there is great demand for prophylaxes or therapies for skin hyperpigmentary disorders. Based on the notion that tyrosinase is a rate-limiting enzyme for melanin biosynthesis, in vitro tyrosinase inhibitor assay screening has been widely utilized in research in this field. However, compounds discovered using this method often display opposite effects. For example, quercetin was identified as a mushroom tyrosinase inhibitor that increased melanin content in cultured mammalian melanocytes and a reconstituted human epidermis model [3,4,5].

In order to identify potential melanogenesis inhibitors, we conducted B16F10 cell-based melanin content assay screening and identified L-765,314 as a potential inhibitor. L765,314 is known as a selective α_1B_-adrenoceptor antagonist [6] and has been used to block noradrenaline and phenylephrine (PE)-induced vasoconstriction [7]. In the present study, we investigated whether L-765,314 reduced melanin accumulation and characterized the molecular mechanism of its action.

## 2. Results

### 2.1. L-765,314 Reduces Melanin Production

**Figure 1:**
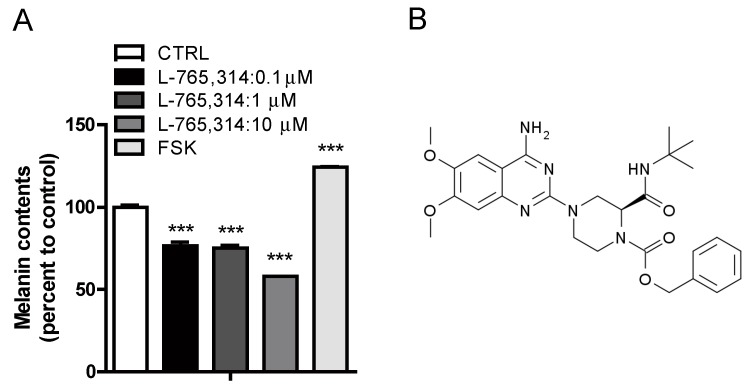
L-765,314 reduced melanin secretion in B16F10 melanoma. (**A**) B16F10 melanoma cells were treated with vehicle or 0.1–10 μM L-765,314. Seventy-two hours after treatment, the melanin secreted from B16F10 cells was examined. Melanin content is given as percent change relative to vehicle-treated controls. (**B**) Chemical structure of L-765,314 (Benzyl (*S*)-4-(4-amino-6,7-dimethoxyquinazolin-2-yl)-2-(tert-butylcarbamoyl) piperazine-1-carboxylate). *** represents *p* < 0.001.

**Figure 2:**
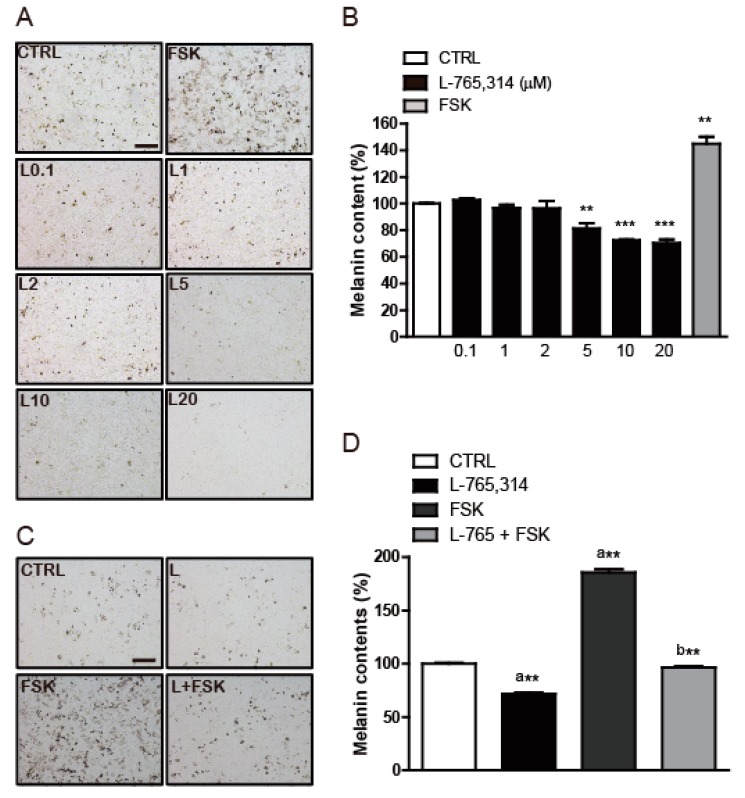
L-765,314 reduced melanin production in normal Mel-ab melanocytes. Ninety-six hours after treatment with 0.1–20 μM L-765,314, (**A**) microscopic images of Mel-ab cells were captured using phase-contrast microscopy and (**B**) melanin content was measured. Forskolin treatment was used as a positive control for melanin production. Mel-ab cells were treated with vehicle, 10 μM forskolin, 10 μM L-765,314, or 10 μM forskolin and 10 μM L-765,314 together for 96 h and (**C**) microscopic images were captured and (**D**) melanin production was compared and presented as percent changes relative to vehicle-treated controls. Statistic test for a compared to control and b compared to forskolin (FSK). Scale bar: 1000 μm, ** and *** represents *p* < 0.01 and *p* < 0.001 respectively. a** represents *p* < 0.01 between control and L765,314 or L765,314 + forskolin treatment, b** represents *p* < 0.01 between forskolin treatment and L765,314 + forskolin treatment.

**Figure 3:**
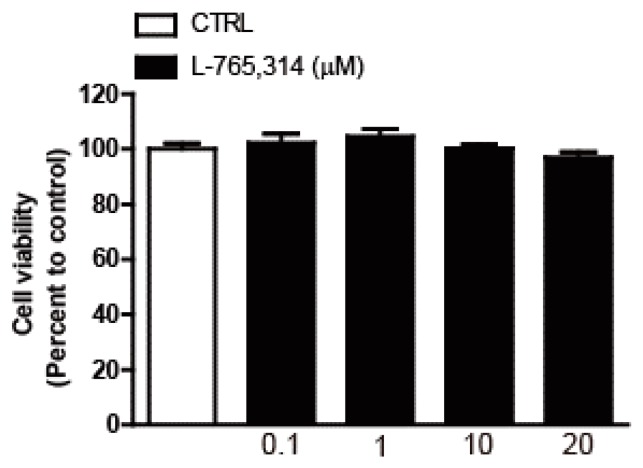
Effect of L-765,314 on cell viability. Mel-ab cells were treated with 0.1–20 μM L-765,314 for 48 h and cell viability was examined by MTT assay.

To discover small-molecule drugs that exhibit anti-melanogenic activity, we conducted high-throughput scale melanin content assay screening. Out of 1280 LOPAC (The library of Pharmacologically Active Compounds) library compounds (Sigmal-Aldrich, St Louis, MO, USA), L-765,314 was one of the compounds that inhibited α-MSH-stimulated melanin secretion without affecting the intracellular ATP levels of B16F10 melanoma cells (Figure S1). After confirming the attenuation of forskolin-stimulated melanin production in B16F10 melanoma cells by L-765,314 (Figure 1A,B), we tested whether the anti-melanogenic effect of L-765,314 was conserved in normal melanocytes. Compared with vehicle treatment, L-765,314 at a concentration of 5 μM reduced melanin accumulation in Mel-ab cells by 19%, and 10 and 20 μM L-765,314 decreased it by 28 and 30%, respectively (Figure 2A,B). Additionally, 10 μM L-765,314 suppressed forskolin-stimulated melanin production in Mel-ab melanocytes (Figure 2C,D). While a dose-dependent decrease in melanin accumulation was observed with L-765,314 treatment, concentrations of L-765,314 up to 20 μM did not affect Mel-ab cell viability, suggesting that the anti-melanogenic effect of L-765,314 was not caused by nonspecific cytotoxicity (Figure 3). Since 10 and 20 μM L-765,314 reduced melanin production comparably without affecting melanocyte cell viability, subsequent experiments were conducted with 10 μM L-765,314.

### 2.2. The Anti-Melanogenic Effect of L-765,314 Is not Associated with α_1B_-Adrenoceptor Signaling

**Figure 4:**
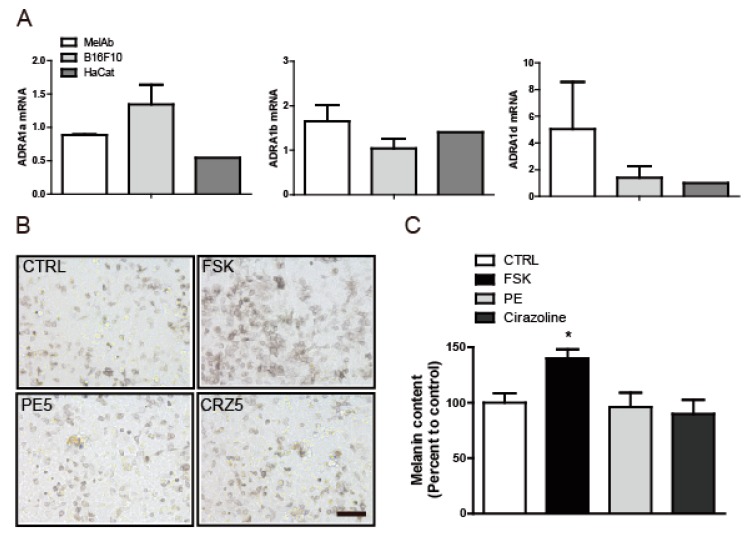
Suppression of melanin production by L-765,314 did not involve the adrenoceptor signaling pathway. (**A**) Expression of ADRA1a, ADRA1b, and ADRA1d in Mel-ab, B16F10, and HaCat cells was analyzed by qRT-PCR. Mel-ab cells were treated with vehicle (DMSO), 10 μM forskolin (FSK), or ADRA agonists, i.e., 5 μM phenylephrine (PE5) and 5 μM cirazoline (CRZ5). Ninety-six hours after treatment, (**B**) microscopic images were captured and (**C**) melanin contents were measured. Melanin contents are given as percent changes relative to vehicle-treated controls. Scale bar: 500 μm, * represents *p* < 0.05.

L-765,314 is a potent, widely used, selective α_1B_-adrenoceptor antagonist. To determine whether the reduction in melanin synthesis induced by L-765,314 was mediated by inhibition of the adrenoceptor signaling pathway [6], we first examined adrenoceptor expression in melanocytes. In mammals, three α_1_-adrenoceptor subtypes, ADRA1a, ADRA1b, and ADRA1d, have been reported and quantitative reverse transcription PCR (qRT-PCR) confirmed that all three subtypes were expressed in melanocytes (Figure 4A). Having observed the expression of ADRA1b, we then considered whether the suppression of melanin production by L-765,314 was mediated by inhibition of adrenoceptors and, conversely, whether the activation of adrenoceptors, or, more specifically, ADRA1b, may enhance melanin production. However, neither exposure to phenylephrine, a selective ADRA agonist [8,9], nor to cirazoline, a full ADRA1a agonist and partial agonist for ADRA1b and ADRA1b [10,11], increased the melanin content in Mel-ab cells (Figure 4B,C and Figure S2).

### 2.3. L-765,314 Downregulates Tyrosinase Activity via Disruption of the PKC Signaling Pathway

**Figure 5:**
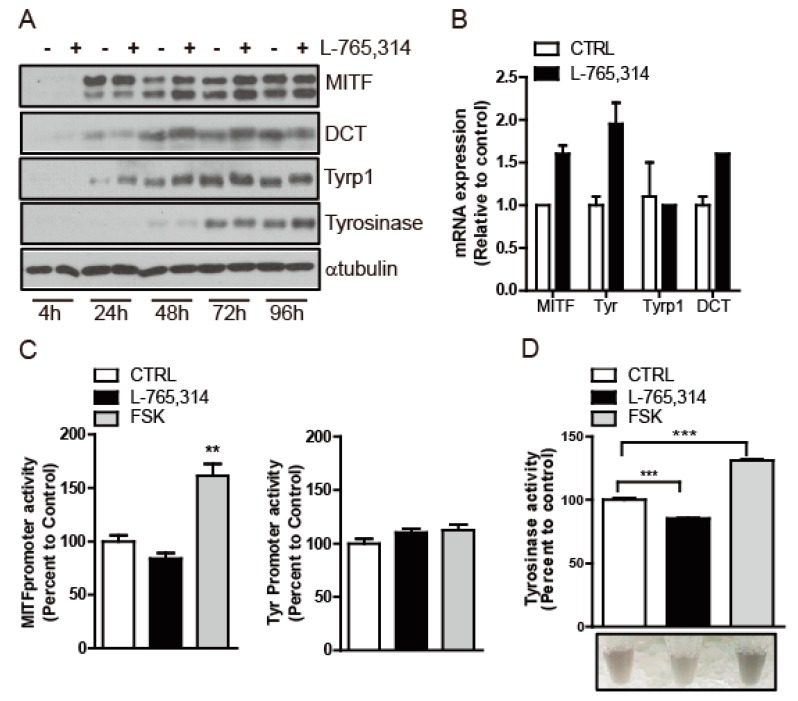
L-765,314 downregulated tyrosinase activity without decreasing tyrosinase expression. (**A**) The expression level of microphthalmia-associated transcription factor (MITF), DCT, Tyrp1, and tyrosinase in Mel-ab cells treated with vehicle and L-765,314 was compared by immunoblotting. α-tubulin was used as an internal loading control. (**B**) The transcript levels of MITF, DCT, Tyrp1, and tyrosinase in L-765,314-treated Mel-Ab cells for 96 h were compared to those of control cells by qRT-PCR. L32 transcript was used as an internal control. (**C**) The effect of L-765,314 on MITF and tyrosinase promoter activity was assessed. Forskolin was used as a positive control for enhancing MITF promoter activity. (**D**) Tyrosinase activity in Mel-ab cells treated with vehicle, L-765,314, or forskolin for 96 h was examined and presented as percent change relative vehicle-treated controls. ** and *** represents *p* < 0.01 and *p* < 0.001 respectively.

To decipher the anti-melanogenic mechanism of L-765,314, we first examined the expression levels of the genes involved in melanogenesis. Mel-ab cells treated with L-765,314 expressed a comparable amount of microphthalmia-associated transcription factor (MITF), DCT, Tyrp1, tyrosinase protein and mRNA to vehicle-treated control Mel-ab cells (Figure 5A,B). Likewise, neither MITF nor tyrosinase promoter activity was suppressed by L-765,314 treatment (Figure 5C). Having seen that L-765,314 does not alter melanogenic gene expression, we then considered the possibility that it is involved with the regulation of tyrosinase activity without altering gene expression. Consistent with the upregulation of tyrosinase expression by forskolin, Mel-ab cells exhibited enhanced tyrosinase activity upon forskolin treatment and downregulated tyrosinase activity upon L-765,314 treatment (Figure 5D).

**Figure 6:**
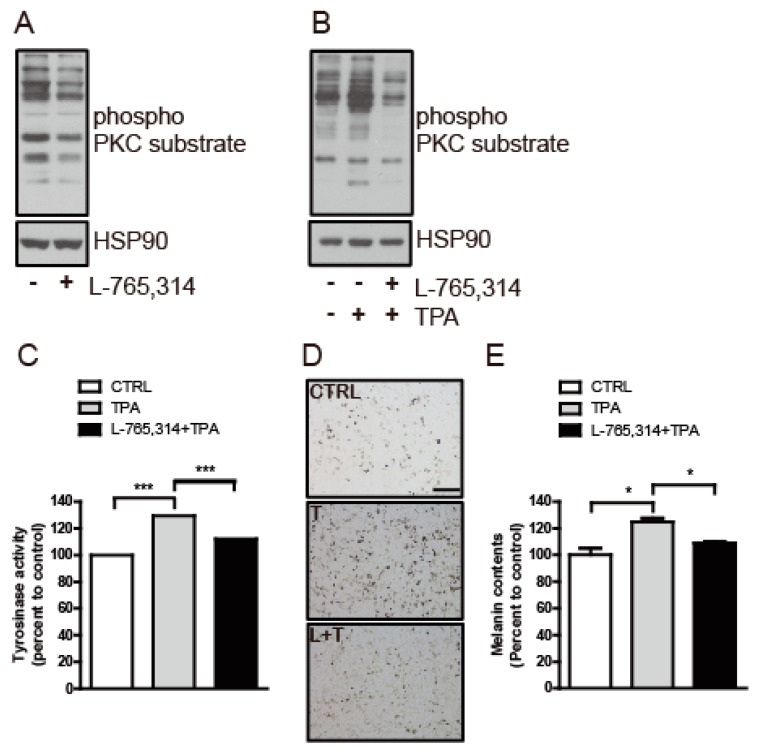
Protein Kinase C (PKC)-stimulated tyrosinase activity was attenuated by L-765,314. (**A**) PKC activity was assessed in Mel-ab cells treated with L-765,314 for 96 h by immunoblotting with phospho-PKC substrate antibody. (**B**) The PKC activity and (**C**) tyrosinase activity of Mel-ab cells treated with vehicle, 12-*O*-Tetradecanoylphorbol 13-acetate (TPA), or L-765,314 and TPA were examined. (**D**) Microscopic images and (**E**) melanin contents of Mel-ab cells treated with vehicle, TPA, or TPA with L-765,314. *, ** and *** represents *p* < 0.05, *p* < 0.01, and *p* < 0.001 respectively.

Multiple signal transduction pathways participate in the regulation of melanogenesis [12,13] and, among these, protein kinase C (PKC) has been shown to regulate tyrosinase activity via direct induction of tyrosinase phosphorylation [14]. To determine whether the L-765,314-mediated downregulation of tyrosinase activity was associated with PKC signaling, the effect of L-765,314 on PKC activity was assessed in Mel-ab cells. Compared with controls, Mel-ab cells treated with L-765,314 maintained lower PKC activity (Figure 6A). It is well-known that 12-*O*-Tetradecanoylphorbol 13-acetate (TPA) stimulates PKC activity in melanocytes and treatment of L-765,314 attenuated TPA-stimulated PKC and tyrosinase activity (Figure 6B,C). Likewise, L-765,314 attenuated TPA-stimulated melanin production in Mel-ab cells (Figure 6D,E).

**Figure 7:**
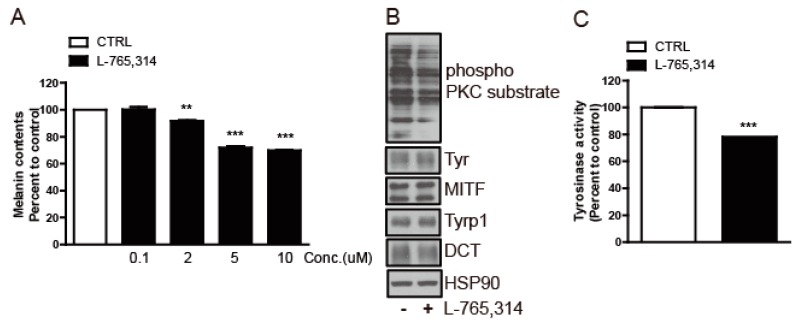
L-765,314 suppressed melanogenesis in normal human melanocytes. (**A**) Melanin contents of normal human melanocyte (NHM) cells treated with 0.1–10 μM L-765,314 for 96 h. (**B**) PKC and (**C**) tyrosinase activity of NHM cells after incubation for 96 h with 10 μM L-765,314. ** and *** represents *p* < 0.01 and *p* < 0.001, respectively.

Finally, to investigate if the anti-melanogenic activity of L-765,314 seen in Mel-ab cells is applicable to human skin, we treated normal human melanocyte (NHM) cells with L-765,314. This treatment reduced the melanin content of NHM cells in a dose-dependent manner, with a 30% decrease in melanin accumulation with 10 μM L-765,314 (Figure 7A and Figure S3). Furthermore, similar to the results observed in Mel-ab cells, L-765,314 also downregulated the tyrosinase activity which accompanied the decreased PKC activity in NHM cells (Figure 7B,C).

## 3. Discussion

Aside from therapeutic applications for skin hyperpigmentary disorders, exponential growth in aesthetic interests demands the development of new, effective, and safe anti-melanogenic agents. According to Global Industry Analysts, the global anti-melanogenic market is projected to reach US $31.2 billion by 2024 [15]. Since tyrosinase is almost exclusively expressed in melanocytes and controls the rate of melanin biosynthesis, the biggest efforts in this field have been attempts to discover melanogenesis inhibitors targeting tyrosinase. Numerous tyrosinase inhibitors have been identified, and hydroquinone, arbutin, and kojic acid, for example, have been utilized both for therapeutic and cosmetic purposes [16]. However, the instability, solubility, and side effects, including irritation, toxicity, and carcinogenicity, of these agents necessitate the development of safer and more effective novel drugs [17,18,19]. To this end, we conducted high-throughput melanin content assay screening. Out of several compounds that reduced α-MSH-stimulated melanin secretion from B16F10 melanoma cells, L-765,314 caught our attention because a simultaneous cellular ATP assay inferred that the suppression of melanin secretion by L-765,314 did not result from the unspecific deterioration of cell viability.

Interestingly, the decrease in melanin production after L-765,314 treatment was not accompanied by a downregulation of melanogenic gene expression; rather, it specifically reduced tyrosinase activity. In regard to tyrosinase activity, a series of studies found that PKC plays a stimulating role in pigmentation. Treatment with the PKC agonist diacylglycerol (DAG), but not a PKC-inactive DAG analog, increased pigmentation in cultured melanocytes and in vivo in a guinea pig model and a blockade of protein kinase activity (PKC, PKG, and PKA) with H-7 suppressed DAG-stimulated melanogenesis [20,21]. Depletion of PKC by chronic exposure to phorbol 12,13-dibutyrate (PDBu) reduced basal and α-MSH-stimulated melanin levels in human melanocytes and murine melanoma cells [22,23]. Park et al. showed that of more than 11 PKC isoforms, PKCα and PKCβ are two major isoforms expressed in human melanocytes [22] and while PKCα expression is ubiquitous in skin tissues including melanocytes, keratinocytes, and fibroblasts, expression of PKCβ is restricted in melanocytes [24,25]. Subsequent studies found a strong correlation between the expression and activity level of PKCβ and melanin content [22], and demonstrated that PKCβ, but not PKCα, was associated with tyrosinase on the outer surface of melanosomes, and regulates tyrosinase activity via direct phosphorylation of serine at tyrosine sites 505 and 509 [14].

While L-765,314 has frequently been used as a selective α_1B_-adrenoceptor inhibitor [6,7], recent studies have shown that this effect could vary depending on the tissues and the presence of additional receptors or targets for L-765,314 [26]. In line with these studies, here we showed that melanin production was not altered by treatment with PE or cirazoline, both α-adrenoceptor agonists, which implies that there may be no direct interaction between α_1B_-adrenoceptor signaling and the regulation of melanin synthesis. Furthermore, we showed that downregulation of tyrosinase activity by L-765,314 was associated with decreased PKC activity as the PKC and tyrosinase activity of basal and TPA-stimulated melanocytes was suppressed by L-765,314, resulting in a reduction in melanin accumulation. Although we have no concrete data regarding the molecular details of PKC inhibition by L-765,314, because pan protein kinase inhibitors are generally characterized as strong apoptotic cell death inducers [27] and L-765,314 did not raise any cytotoxicity issues, our data suggest that L-765,314, instead of having a broad range of protein kinase targets, might exhibit specific activity on certain subtypes of PKC, possibly PKCβ.

In conclusion, we found that L-765,314 reduced melanin production by targeting the PKC-dependent regulation of tyrosinase activity. In line with our studies, bisindolylmaleimide, which showed a higher selectivity for PKCβ over other PKC isoforms, reduced melanin accumulation via prevention of TPA-stimulated phosphorylation and the activity of tyrosinase [28,29,30]. The effect of melanin precursors, l-tyrosine and l-DOPA, on melanin synthesis was reported previously [31]. While inhibition of tyrosinase activity by L-765,314 may raise the intracellular level of melanin precursors, accumulated melanin precursors might be simply degraded or alternatively enter the pheomelanin synthesis pathway. In fact, pheomelanin synthesis is the default pathway while eumelanin synthesis requires both high levels of l-tyrosine and tyrosinase activity [32,33]. Understanding the molecular mechanism of PKC inhibition by L-765,314 and verifying its anti-melanogenic activity in in vivo models should be topics of further study to determine whether L-765,314 could be applied to treat human hyperpigmentary disorders. Finally, our study also suggests that targeting PKC could be a promising strategy to develop effective and safe depigmenting agents beyond direct tyrosinase inhibitors [13].

## 4. Materials and Methods

### 4.1. Chemical Reagents

The LOPAC library (Sigma-Aldrich, St Louis, MO, USA) was used for B16F10-based melanin content screening. L-765,314 and TPA were purchased from Sigma-Aldrich (St Louis, MO, USA). Forskolin (FSK) and cirazoline hydrochloride (CRZ) were purchased from Tocris Bioscience (Bristol, UK) and phenylephrine hydrochloride (PE) was purchased from Tokyo Chemical Industry (Tokyo, Japan).

### 4.2. Cell Culture

HEK-293T cells and B16F10 murine melanoma cells (The Korean Cell Line Bank, Seoul, Korea) were cultured in Dulbecco’s Modified Eagle Medium (DMEM) with 10% fetal bovine serum (FBS) (Corning, Corning Life Sciences, NY, USA) and 1% P/S. Mel-ab cells from a mouse-derived spontaneously immortalized melanocyte cell line were maintained in DMEM supplemented with 10% FBS (Corning, Corning Life Sciences, NY, USA), 100 nM TPA (Sigma-Aldrich, St. Louis, MO, USA), 1 nM cholera toxin (Sigma-Aldrich), and 1% P/S. NHM obtained from Invitrogen (Carlsbad, CA, USA) were maintained in Medium 254 (Invitrogen) containing a human melanocyte growth supplement (Invitrogen). All cells were maintained in a humid environment at 5% CO_2_.

### 4.3. Screening

For melanin content assay-based melanogenesis inhibitor screening, B16F10 melanoma cells were plated in 96-well plates at 1 × 10^4^ cells/well in phenol red-free DMEM (Gibco-BRL, Waltham, MA, USA), with 10% FBS and 1% P/S. Twenty-four hours after plating, the cells were treated with vehicle, arbutin (100 μg/mL; negative control), α-MSH alone (500 nM; positive control), or α-MSH (500 nM) with 0.1, 1, or 10 μM library compounds, respectively. Seventy-two hours after treatment, the melanin contents of the culture media collected were measured and compared. To distinguish the specific inhibitory effect of melanogenesis-related chemicals from any unspecific toxic effects, we performed a cell proliferation assay (CellTiter-Glo Assay, Promega Corp., Madison, WI, USA) with the remaining B16F10 cells and excluded chemicals that reduced cell viability by more than 10% relative to the vehicle treatment.

### 4.4. Cell Viability

Cell viability was assessed using an MTT assay kit (Duchefa-biochemie, Haarlem, Netherlands) according to the manufacturer’s instructions. Mel-ab cells cultured in 24-well plates at 1.5 × 10^5^/well were exposed to 0.1–20 μM L-765,314. Forty-eight hours after treatment, MTT was added to the culture media to produce 1 mg/mL MTT solution and this was incubated for another 1 h under culture conditions. Mel-ab cells were then washed with phosphate buffer saline (PBS) and intracellular MTT was dissolved with 150 μL DMSO, then optical density was measured by reading absorbance at 562 nm using a microplate reader (BioTek, Winooski, VT, USA). Cell viability was given as percent changes relative to controls.

### 4.5. Tyrosinase Activity

Mel-ab cells were plated on 6-well plates at a density of 6 × 10^5^ cells/well in DMEM supplemented with 10% FBS and 1% P/S (without TPA and cholera toxin). The cells were stabilized overnight then treated with L-765,314 (10 μM), FSK (10 μM), TPA (100 nM), or a combination as indicated in the figures, and the drug-containing culture media was replaced every other day for 4 days. At 96 h, the cells were washed with cold PBS and lysed in 300 μL of tyrosinase lysis buffer (phosphate buffer, pH 6.8, containing 1% Triton X-100) with repeated freeze/thaw cycles and centrifuged at 15,000 rpm at 4 °C for 10 min. Then, 90 μL supernatant mixed with 10 μL of 10 mM l-DOPA in tyrosinase lysis buffer was incubated at 37 °C, and tyrosinase activity was measured by reading the absorbance at 475 nm using a microplate reader (BioTek). Tyrosinase activity was normalized to the amount of protein used and was given as percent change relative to the vehicle-treated controls.

### 4.6. Antibodies and Western Blots

Mel-ab cells were washed with cold PBS and lysed in 10 mM Tris (pH 7.4) containing 5 mM EDTA and 1% SDS followed incubation at 98 °C for 5 min. Protein samples separated by SDS PAGE were transferred to a nitrocellulose membrane (MilliporeSigma, Burlington, MA, USA or ATTO Technology, Amherst, NY, USA), blocked with TTBS containing 3% BSA, and subjected to immunoblotting. Tyrosinase, Tyrp1, and DCT antibodies were purchased from Santa Cruz Biotechnology (Dallas, TX, USA) and MITF was purchased from Neomarkers (Fremont, CA, USA). HSP90 (SantaCruz Biotech) was used as an internal loading control.

### 4.7. RNA and qRT-PCR

Total RNA from Mel-ab, B16F10, and HaCat cells cultured in 24-well plates was isolated using FavorPrep™ Tri-RNA Reagent (FAVORGEN^®^, Changzhi Township, Taiwan). For first strand cDNA synthesis, 500 ng isolated total RNA was reverse transcribed using a ReverTra Ace^®^ qPCR RT Kit (Toyobo, Osaka, Japan) following the manufacturer’s instructions. mRNA expression was examined by qRT-PCR using a Lightcycler480 (Roche Applied Science, IN, USA) with THUNDERBIRD™ SYBR^®^ qPCR Mix (Toyobo, Japan). qRT-PCR was performed with initial denaturation at 94 °C for 3 min followed by 45 cycles of 94 °C for 15 s, 60 °C for 30 s, and 72 °C for 20 s. The L32 of β-actin expression was used as a reference. Specific primer sets used for the amplification of each gene are presented in Table S1.

### 4.8. Promoter Activity Assay

The effect of L-765,314 on MITF and tyrosinase promoter activity was assessed as described previously [34]. Briefly, 494 bp of the 5′ flanking sequence of the *MITF* promoter and 390 bp of the 5′ flanking sequence of the *TYR* promoter cloned into pGL3 were transfected to HEK-293T cells. Twenty-four hours after transfection, transfected cells were treated with either vehicle (DMSO) or 10 μM L-765,314 for another 6 h and luciferase activity was measured.

### 4.9. Statistics

The data are presented as means ± S.E.M., and statistical significance was determined by an unpaired Student’s *t*-test using GraphPad Prism software (version 5.01). In this study, *p* < 0.05, *p* < 0.01, and *p* < 0.001 were considered statistically significant and are represented by *, **, and ***, respectively.

## Supplementary Materials

The following are available online, Figure S1: Melanin content assay-based HTS screening in B16F10 cells. Following 500 nM α-MSH treatment, 0.1, 1, or 10 μM L765,314 was applied and melanin content was measured, Figure S2: Mel-ab cells were treated with vehicle (DMSO), 5 μM forskolin (FSK), or 15 μM phenylephrine (PE15) and 15 μM cirazoline (CZ15). Ninety-six hours after treatment, (A) microscopic images were captured and (B) melanin contents were measured. Melanin contents are given as percent changes relative to vehicle-treated controls. Scale bar: 1000 μm, Figure S3: Microscopic images of normal human melanocytes treated with 0.1–10 μM L-765,314 for 96 h. Scale bar: 200 μm, Table S1: Primer list for qRT-PCR.

## Abbreviations

UVR	Ultraviolet Radiation
FSK	Forskolin
MEFs	Mouse Embryonic Fibroblasts
MITF	Microphthalmia-Associated Transcription Factor
Tyr	Tyrosinase
Tyrp1	Tyrosinase-Related Protein 1
DCT	Dopachrome Tautomerase
l-DOPA	l-Dihydroxyphenylalanine
PKC	Protein kinase C
TPA	12-*O*-Tetradecanoylphorbol 13-acetate
