# Supplementary material for: L-765,314 Suppresses Melanin Synthesis by Regulating Tyrosinase Activity
Source: Molecules. 2019 Feb 21;24(4):773. doi: 10.3390/molecules24040773 (PMC6412649; doi:10.3390/molecules24040773)
Supplement: Supplementary file 1 [file molecules-24-00773-s001.pdf]

Supplementary Materials:

Table S1. Primer list for qRT-PCR.

Gene	Forward primer	Reverse primer
L32	5′-AGATCCTGATGCCCAACATC-3′	5′-CAGCTCCTTGACATTGTGGA-3′`
mADRA1a	5′-GCTCCGTATCCACCGTAAAA-3′	5′-TCTCTCGGGAAAACTTGAGC-3′
mADRA1b	5′-GAGGCTGCGCTTACACCTAC-3′	5′-CTGCCACTGTCATCCAGAGA-3′
mADRA1d	5′-TCCGTAAGGCTGCTCAAGTT-3′	5′-GGACGAAGAAAAAGGGGAAC-3′
hADRA1a	5′-ACATTTCCAAGGCCATTCTG-3′	5′-CAGGCTACGGAGAGGATCAC 3′
hADRA1b	5′-TCTCCAGGGAAAAGAAAGCA-3′	5′-GCGGTAGAGCGATGAAGAAG-3′
hADRA1d	5′-CTCAGAGGTGGAGGCTGTGT-3′	5′-TATCGGTCTCCCGTAGGTTG-3′
MITF	5′-TGAAGCAAGAGCATTGGCTA-3′	5′-TCCACAGAGGCCTTGAGAAT-3′
Tyrosinase	5′-TTATGCGATGGAACACCTGA-3′	5′-ACTGGCAAATCCTTCCAGTG-3′

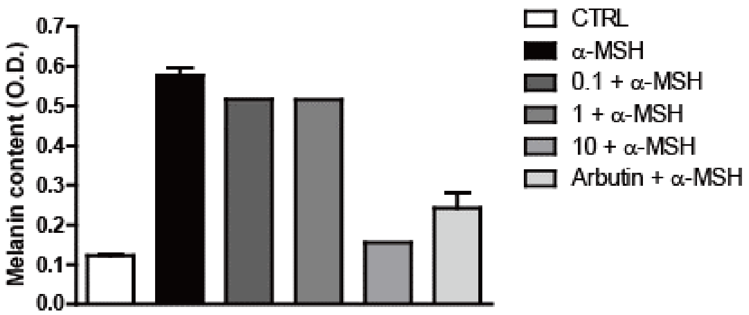

Figure S1. Melanin content assay-based HTS screening in B16F10 cells. Following 500 nM α-MSH treatment, 0.1, 1, or 10 μM L765,314 was applied and melanin content was measured.

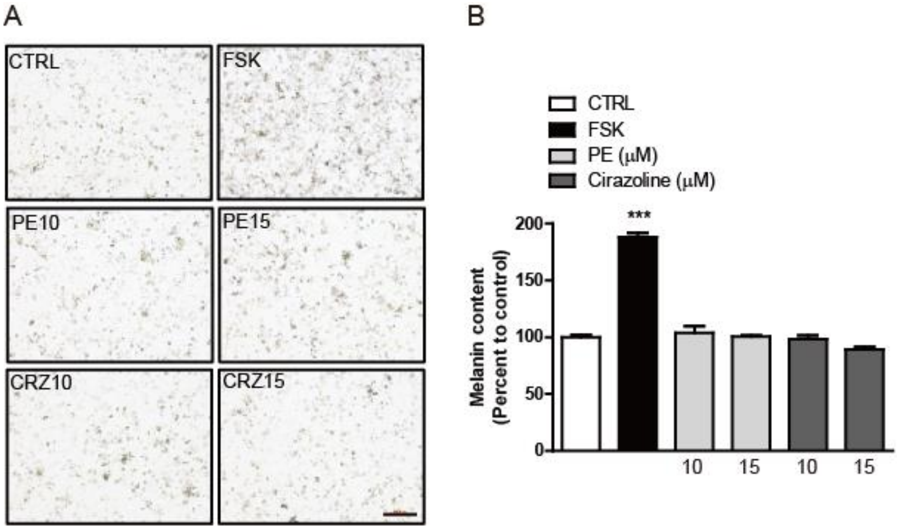

Figure S2. Mel-ab cells were treated with vehicle (DMSO), 5 μM forskolin (FSK), or 15 μM phenylephrine (PE15) and 15 μM cirazoline (CZ15). Ninety-six hours after treatment, (**A**) microscopic images were captured and (**B**) melanin contents were measured. Melanin contents are given as percent changes relative to vehicle-treated controls. Scale bar: 1000 μm.

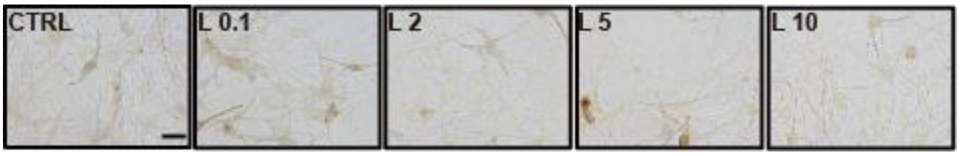

Figure S3. Microscopic images of normal human melanocytes treated with 0.1–10 μM L-765,314 for 96 h. Scale bar: 200 μm.
